# Artificial intelligence for precision gene therapy: emerging technologies, clinical applications, and future directions

**DOI:** 10.3389/fgene.2026.1940629

**Published:** 2026-09-24

**Authors:** Govinder Flora, Abhinay Thakur

**Affiliations:** Division of Research Connect, Lovely Professional University, Phagwara, Punjab, India

**Keywords:** artificial intelligence, biomanufacturing, CRISPR, gene therapy, genomics

## Abstract

Artificial Intelligence (AI) is revolutionizing gene therapy from an empirical and iterative process to a predictive, data-driven and increasingly intelligent therapeutic development approach. This review covers the application of AI extensively throughout the gene therapy development process, from target therapeutic discovery, identification of disease genes, variant interpretation, integration of multi-omics data, optimization of CRISPR, engineering of the vector, prediction of immunogenicity, manufacturing, and clinical development and post-marketing surveillance. The role of machine learning, deep learning, foundation models, generative AI and digital twins for advancing therapeutic design, boosting vector performance, manufacturing optimization, patient stratification and enabling precision medicine is discussed. Lastly, we provide directions for future research and highlight the need for close multidisciplinary cooperation between computational scientists, biologists, clinicians, industry, and regulatory agencies to facilitate the development of safe, effective and personalized genetic medicines responsibly.

## Graphical Abstract

**Figure:**
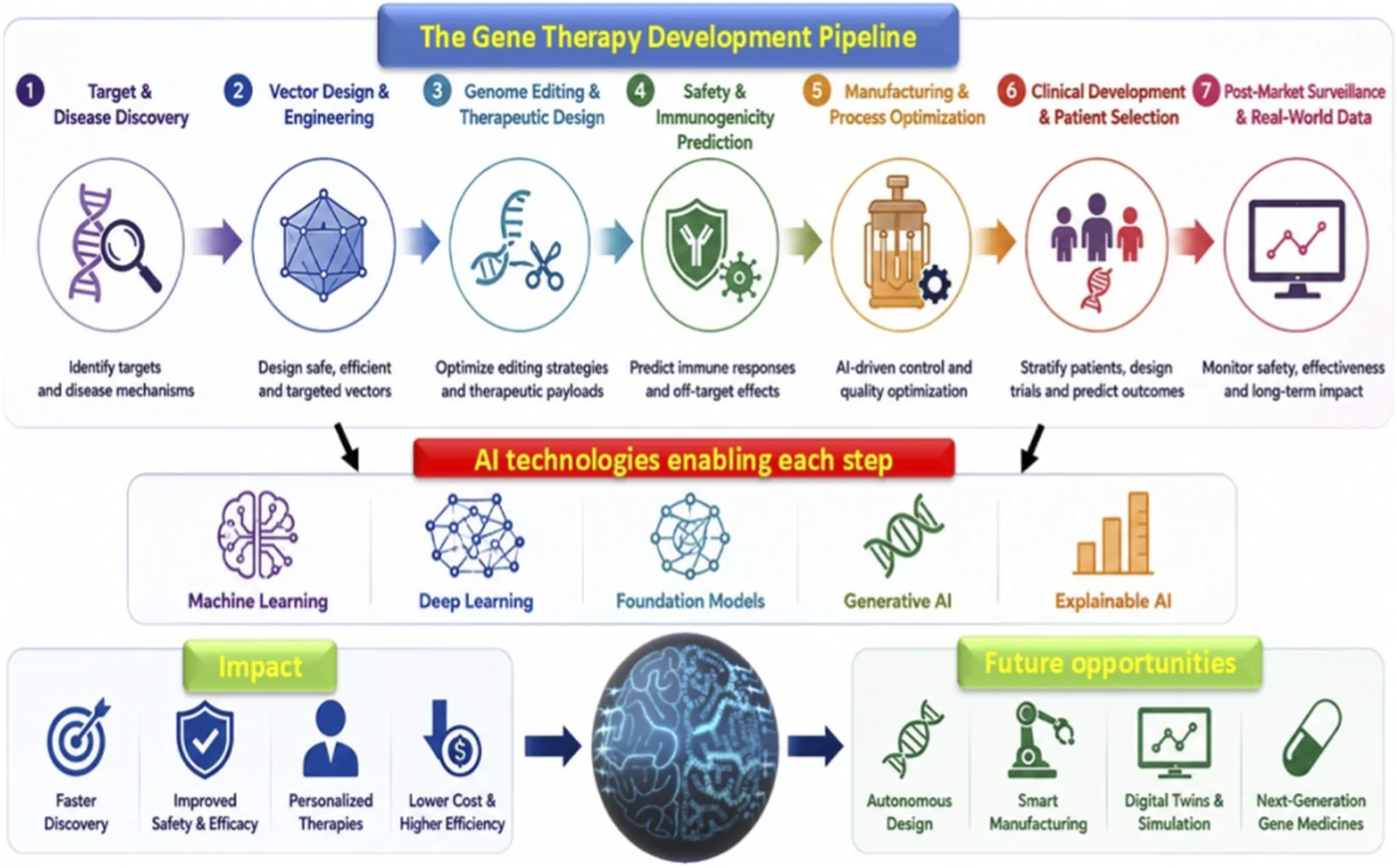

## 1 Introduction

Over the last few years, AI has shown tremendous promise in advancing scientific research across various fields, such as drug discovery, protein engineering, structural biology, genomics, medical imaging, and personalized medicine, with the potential to significantly shorten research and development timelines, enhance prediction models, and boost both the rate and efficacy of drug development (Lebedev et al., 2020; Li et al., 2023). Examples of protein structure prediction using AlphaFold and recent genomic foundation models have shown the ability of AI to solve longstanding problems in biology and created new avenues for the rational design of next-generation therapeutics. Over the years, viral vector technologies and genome editing (e.g., CRISPR-Cas systems, base editing, prime editing and non-viral delivery platforms) have broadened therapeutic applications of gene therapy (Dawoodbhoy et al., 2021; Sanchez Bolaños et al., 2021; Combi et al., 2022). Such technologies have been applied to the successful treatment of a few otherwise untreatable diseases such as inherited retinal diseases, spinal muscular atrophies, β-thalassemia, sickle cell disease, hematological malignancies and hemophilia via chimeric antigen receptor T-cell (CAR-T) therapies. Further validation of the clinical feasibility of genetic medicines has been seen in the regulatory approval of several gene therapy products across the United States, Europe and Asia, which has shown the potential for genetic medicines to fundamentally change the management of rare and complex diseases. Figure 1 shows the AI applications across the gene therapy development pipeline, highlighting AI-driven advancements in therapeutic target discovery, vector engineering, genome editing, manufacturing, preclinical evaluation, clinical development, and post-marketing surveillance.

**FIGURE 1:**
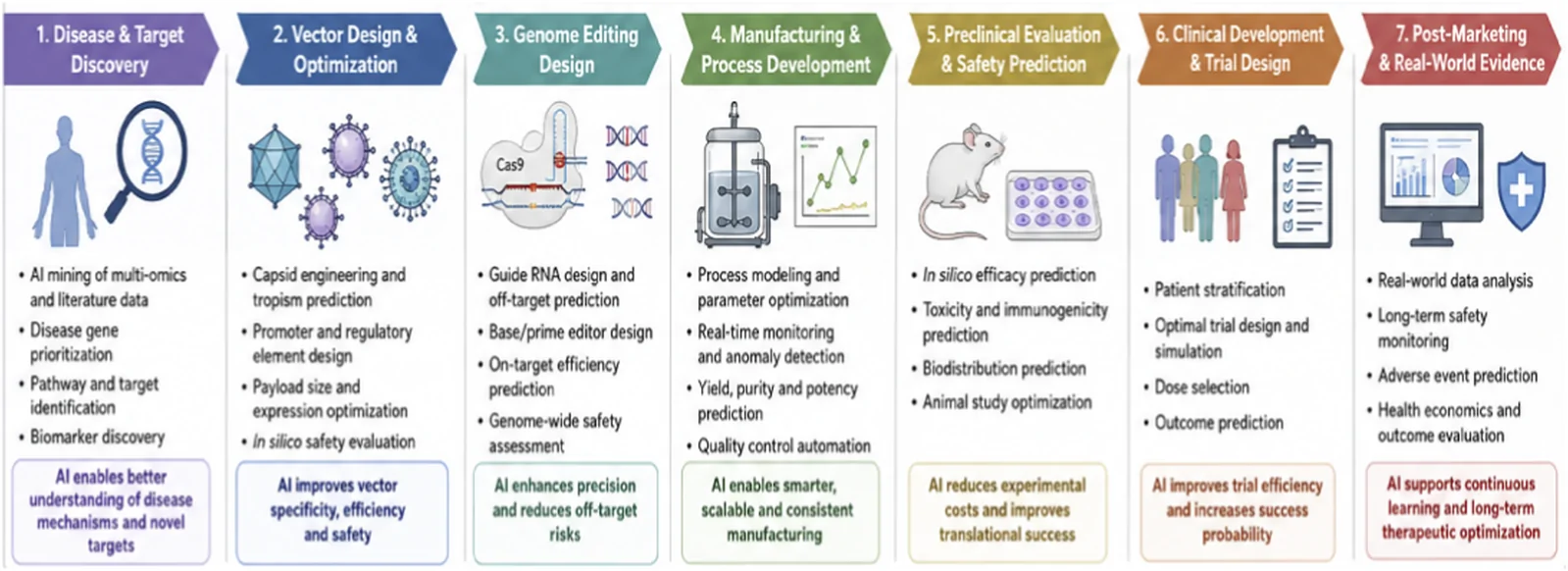
Schematic illustration of the role of artificial intelligence in accelerating gene therapy development from disease target identification to real-world clinical implementation.

Nevertheless, the development of gene therapies is still a very complicated, multi-disciplinary and resource-intensive process. However, a successful gene therapy must rely on the accurate identification of disease-causing genes, complete characterization of pathogenic genetic variants, optimization of genome editing strategies, the engineering of highly efficient genome delivery methods, prediction and minimization of immune responses, scalable and cost-effective manufacturing, careful patient stratification, clinical trial design, and long-term post-marketing surveillance (Eti et al., 2025). The worldwide research efforts also underscore the growing significance of AI in the field of gene therapy. In the United States, partnerships between academic institutions, biotechnology firms and the regulatory agencies have propelled the acceleration of genomic interpretation, rare disease diagnosis, and therapeutic target discovery in precision medicine programs through the use of AI (Goertzel, 2007; Kaplan et al., 2021; Lindegger et al., 2022). Large-scale sequencing programs have provided valuable data sets that allow increasingly accurate prediction of disease-associated variants and therapeutic responses using AI. The UK has invested heavily in genomic medicine and population-scale sequencing projects have built an extensive infrastructure for the application of machine learning to disease prediction, patient stratification, and personalized therapeutic development. Table 1 lists global initiatives driving artificial intelligence-enabled gene therapy development. The global initiatives highlight the growing recognition and acceptance of AI-driven gene therapy as a key area of scientific focus, with significant potential for impact in future healthcare systems.

**TABLE 1:** Global initiatives driving artificial intelligence-enabled gene therapy development.

Region	Major AI initiatives in gene therapy	Representative outcomes and future impact
United States	Integration of AI with genomics, precision medicine, rare disease diagnosis, and therapeutic target discovery through collaborations among academic institutions, biotechnology companies, healthcare organizations, and regulatory agencies	Accelerated genomic interpretation, AI-assisted pathogenic variant prediction, personalized therapeutic development, improved target discovery, and faster clinical translation of gene therapies
United Kingdom	Population-scale genomic sequencing programs integrated with machine learning, electronic health records, and precision medicine initiatives to support disease diagnosis and treatment optimization	Enhanced disease risk prediction, patient stratification, individualized therapeutic selection, improved genomic medicine infrastructure, and data-driven precision healthcare
European Union	Collaborative research integrating AI with synthetic biology, systems biology, advanced biomanufacturing, and regulatory science to develop next-generation gene therapies	Accelerated therapeutic innovation while addressing explainable AI, data governance, ethical considerations, regulatory compliance, and standardized clinical implementation
China	AI-driven biotechnology supported by large-scale genomic databases, high-performance computing, and national investments in precision medicine and genome engineering	Rapid advances in genome editing, vector optimization, therapeutic target discovery, precision medicine research, and accelerated development of AI-assisted genetic therapies
Japan	National precision medicine initiatives utilizing AI for genomic analysis, disease diagnosis, clinical decision support, and translational biomedical research	Improved molecular diagnostics, personalized therapeutic strategies, enhanced translational medicine, and integration of AI into routine clinical practice
Singapore	National programs promoting AI-enabled genomic medicine, biomedical informatics, translational healthcare, and precision medicine research	Strengthened precision medicine infrastructure, improved genomic analytics, enhanced clinical decision support, and accelerated translation of AI-driven genetic medicine into healthcare systems

In this fast-changing scientific environment, the current review offers a thorough overview of the growing importance of artificial intelligence in the intelligent development of gene therapies. It critically reviews the use of AI technologies across the entire therapeutic development process from disease gene discovery to multi-omics integration and genome editing, vector bioengineering, prediction of immunogenicity, manufacturing optimization, clinical development and post-marketing surveillance. In addition to discussing emerging technologies like generative AI, biological foundation models, digital twins, autonomous laboratories, and intelligent biomanufacturing, the review also highlights existing barriers to translation, challenges, and research opportunities.

Although several recent reviews have addressed artificial intelligence within individual segments of the gene therapy pipeline—such as genomic variant interpretation, CRISPR guide design, or viral vector engineering considered in isolation—few have systematically examined how AI-driven methods interconnect across the entire development continuum, from target discovery through manufacturing to post-marketing surveillance. Prior reviews have also tended to describe individual studies rather than critically compare methodologies, evaluate the strength of the underlying evidence, or identify where AI-driven claims currently outpace validated clinical outcomes. The present review seeks to address this gap by explicitly linking AI applications across sequential pipeline stages, by critically appraising the maturity and limitations of the evidence base at each stage, and by consolidating translational barriers that must be overcome for AI-enabled gene therapies to reach routine clinical practice.

## 2 Fundamentals of gene therapy

### 2.1 Principles of gene therapy

Gene therapy is one of the most transformative biomedical developments of recent times, with the potential to tackle diseases at their source and not just the symptoms. The main distinction of gene therapy from conventional pharmacological therapies is that the latter are typically directed at further downstream pathological mechanisms or physiological consequences, whereas gene therapy aims at correcting the primary molecular defect involved in the development of the disease (Sadeghi et al., 2024; Xu et al., 2025). The development of gene therapy has been further advanced by the structure of DNA being elucidated, advances in recombinant DNA technology, completion of the Human Genome Project, the development of high-throughput sequencing technologies and the emergence of programmable genome-editing systems. Initial clinical studies in the 1990s showed the enormous promise and the many hurdles of genetic interventions (Singh et al., 2023; Aboti et al., 2026). Early clinical trials revealed that some vectors caused immune-mediated toxicity and insertional mutagenesis, which highlighted the need for safer vectors, better delivery methods and more precise genome-editing technologies. To fast and accurately make an intraoperative diagnosis of uterine disease, Chen et al. (2025) created an intelligent molecular diagnostic system by combining Raman molecular fingerprint imaging with artificial intelligence. The study employed Raman spectroscopy to collect label-free molecular vibration-specific data from frozen tissue sections of uterine fibroids, adenomyosis, endometrial polyps, and endometrial carcinoma that yielded a set of 2,364 high dimensional spectral data from 140 clinical cases (Figure 2). For more accurate diagnostics, the authors used Principal Component–Linear Discriminant Analysis (PCA-LDA) and Convolutional Neural Networks (CNNs) while, at the same time, integrating several machine learning algorithms such as Support Vector Machine (SVM) and Random Forest in a dynamic fusion decision-making. This multimodal AI approach allowed for effective discrimination between the four different uterine diseases and also made diagnosis possible within about 5 minutes, thus supporting clinical decision-making intraoperatively. The proposed framework, combining molecular spectroscopy and deep learning, was able to address several drawbacks of traditional frozen section histopathology, such as observer dependency and delayed diagnosis. It should be noted that this study addresses intraoperative molecular diagnostics rather than a core gene therapy process; it is discussed here only to illustrate the classification and multimodal-fusion capabilities (CNN, SVM, PCA-LDA) that are later applied to gene therapy-specific problems such as vector tropism prediction and patient stratification, and readers should not interpret it as direct evidence for gene therapy applications.

**FIGURE 2:**
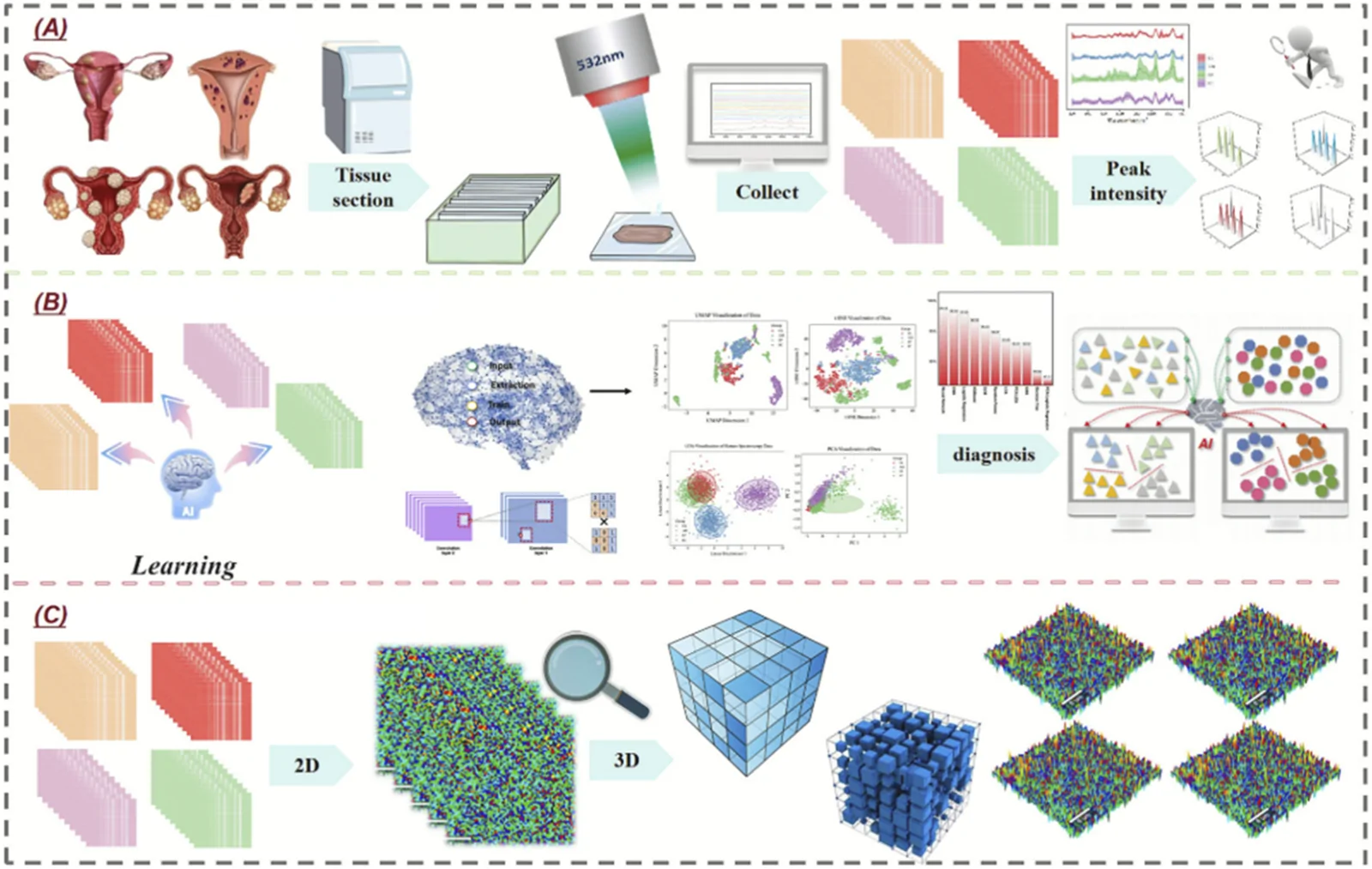
Workflow of the Raman-deep learning diagnostic system. Fresh intraoperative tissues from four uterine diseases (n = 140 cases) were analyzed to build a molecular vibrational database (2364 Raman spectra, 400–1800 cm^−1^). A hybrid PCA-LDA/CNN architecture enabled spatial-spectral analysis, dynamically inte-grating 11 machine learning algorithms for accurate classification. The system achieved 5 min diagnosis via label-free detection, pioneering a shift from morpho-logical to molecular vibration-based diagnostics (Chen et al., 2025). **(A)** Spectral collection **(B)** Artificial intelligence diagnosis **(C)** Disease imaging visualization.

Sankaradass and Manish (2026) presented a quantum-inspired predictive healthcare framework, which will combine multimodal artificial intelligence with blockchain technology for the purpose of enhancing personalized oncology. It integrates heterogeneous patient information such as genomics, clinical, medical imaging, and lifestyle data, and applies sophisticated multimodal data fusion methods based on deep learning to enhance cancer prediction and personalized treatment planning. The framework also supports the use of quantum-inspired optimization algorithms, such as the Variational Quantum Classifier (VQC) and Quantum Approximate Optimization Algorithm (QAOA), which allow models to converge more quickly and make more accurate predictions than standard AI models. In addition, a Blockchain-based data governance layer was added to ensure secure, decentralized, and tamper-proof data management of sensitive healthcare information, enabling transparent data sharing and collaboration between healthcare institutions, laboratories, and clinical stakeholders while maintaining patient privacy. The hybrid edge-cloud architecture is scalable and low-latency for real-time clinical decision support, and enables practical clinical applications in healthcare. Experimental results showed better prediction accuracy, inference speed and data security compared to the conventional AI-based oncology systems. The study underscores the promise of integrating quantum-inspired AI, multimodal data integration, and blockchain technologies to create secure and intelligent clinical decision-support platforms. While its primary focus is on oncology, the proposed framework offers much to consider for future AI-powered precision medicine ecosystems that could be applied to personalized gene therapy, patient stratification, treatment optimization and the secure management of genomic and clinical data. We include this framework only as an illustration of secure, multimodal data-fusion architecture; quantum-inspired optimization and blockchain data governance have not yet been directly validated in a gene therapy context, and their inclusion here should not be read as evidence that these technologies are established components of gene therapy development.

### 2.2 Therapeutic strategies

The advent of various therapeutic approaches targeting different molecular pathways involved in human diseases has been an important driving force for the remarkable progress of gene therapy. Gene therapy does not usually target downstream pathological pathways as is standard with pharmacological therapy, but acts directly at the level of the gene by altering, replacing, suppressing, or repairing faulty genes that play a role in disease onset and/or progression. No single therapeutic approach is broadly applicable to genetic disorders, as they have many different molecular abnormalities such as loss-of-function mutations, gain-of-function mutations, chromosomal rearrangements, dysregulated expression of genes and acquired somatic mutations (Gu et al., 2026). Instead, several complementary approaches have been developed that are specific to the biological nature of particular diseases and therapeutic targets. These innovations, combined with recent progress in molecular biology, synthetic biology, genome engineering and artificial intelligence, have opened the door to increasingly precise, efficient and personalized genetic interventions. To facilitate the design of lung cancer-specific receptors and the intelligent optimization of sensing interfaces for early detection of cancer, Yu et al. (2026) put forward an innovative framework that combines chemical sensors with a generative artificial intelligence (AI) model. In this study, electrochemical sensing by the nanocomposite materials and optical enhancement detection platform were integrated to realize ultrasensitive detection of lung cancer biomarkers. Multimodal generative AI models were used to analyze multi-omics datasets and to design intelligent receptors that are more topologically adaptable to achieve better molecular recognition, which in turn boosts biomolecular binding efficiency and diagnostic accuracy. The proposed sensing platform (Figure 3), which uses AI, proved effective in early detection of lung cancer, which could lead to better health outcomes for patients by enhancing the accuracy of disease detection and personalized treatment strategies. In addition, the use of portable sensing devices and wearable monitoring technologies further improved the translatability of the system, enabling low-cost and real-time diagnostics, especially in resource-limited healthcare environments. The authors also highlighted key challenges such as the explainability of generative AI models, the need for chemical sensor environmental stability and solid validation prior to clinical use. The possibilities of future development, namely, biomimetic adaptive sensing and quantum-biological interface integration, were suggested for further improvement of intelligent diagnostic systems. Overall, the findings of this study underscore the potential of generative AI in precision medicine, pointing to the future direction of AI-enabled molecular diagnostics and targeted therapeutic interventions. It is worth noting explicitly that this example, like several others available in the current literature, concerns AI-enabled disease diagnostics rather than the *de novo* discovery of druggable or gene-editable therapeutic targets; studies applying generative or multimodal AI directly to therapeutic target identification for gene therapy remain comparatively scarce, and this asymmetry in the evidence base is itself a limitation worth highlighting for the field.

**FIGURE 3:**
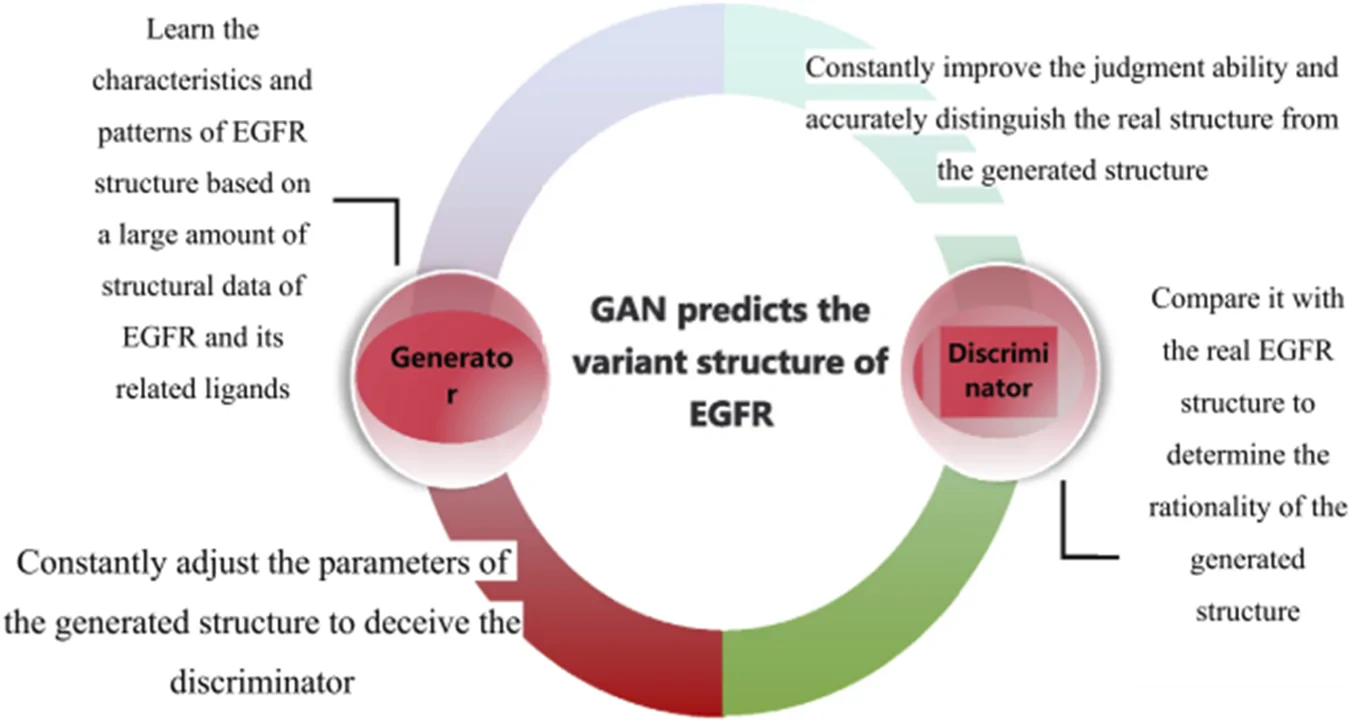
The GAN model predicts the structural variation in epidermal growth factor receptor (EGFR) (Yu et al., 2026).

### 2.3 Gene delivery systems

For gene therapy to become a useful tool for treating human diseases, appropriate therapeutic targets must be identified, effective genetic agents must be developed and therapeutic nucleic acids must be delivered into the target cells safely, efficiently and selectively. However, therapeutic genes, messenger RNA (mRNA), small interfering RNA (siRNA), antisense oligonucleotides and genome-editing components have large molecular sizes and are hydrophilic and susceptible to enzymatic degradation, which means they cannot readily cross the cellular membrane and therefore are the backbone of modern gene delivery systems in gene therapy (Luer et al., 2026). Naked nucleic acids entering the body are quickly broken down by nucleases in the circulation and rapidly excreted by the kidneys, as well as being removed by the immune system before they can reach the target tissues. These factors make it crucial to use specialized delivery platforms to ensure the protection of therapeutic cargo, delivery to the cell, endocytosis, and retention of the biological activity within the cell. An ideal gene delivery system should be efficient, tissue-specific, have minimal immunogenicity, have a large capacity for carrying a therapeutic gene, be scalable for manufacture, and be highly safe while maintaining stable therapeutic gene expression. Although great advances have been made in technology, no one delivery system meets all of these criteria, and delivery system engineering is one of the most dynamic areas of gene therapy research. Gene delivery systems can be generally divided into viral and non-viral platforms, with their own unique biological properties, benefits and drawbacks. Viral vectors are genetically engineered viruses that have pathogenic viral components removed, but retain the natural viral evolution potential to introduce genetic material into host cells (Holzinger et al., 2023; Yosboon et al., 2024). The transduction efficiency of these vectors is very high, and has made them the go-to delivery system for many clinically approved gene therapies. On the other hand, the non-viral delivery systems utilize synthetic nanoparticles, lipids, polymers, peptides, extracellular vesicles, and inorganic nanomaterials to deliver genetic materials without depending on the mechanism of viral infection. While gene transfer efficiency remains superior with viral vectors, non-viral systems are making rapid strides in nanotechnology, biomaterials science, and artificial intelligence, closing the performance gap for these systems, while also providing safety and manufacturing flexibility benefits.

Recent years have also seen rising interest in inorganic nanoparticles, such as gold nanoparticles, silica nanoparticles, magnetic nanoparticles, calcium phosphate particles and carbon-based nanomaterials, for gene delivery applications. These materials have special physicochemical properties, such as magnetic field, ultrasonic, and light responsiveness; imaging; high loading capacity; and tunable surface chemistry. The theranostic gene delivery as a new emerging field has been enabled by multifunctional nanocarriers with the dual function of both therapeutic gene delivery and diagnostic imaging. For instance, magnetic nanoparticles have been used for image-guided delivery of therapeutic nucleic acids to the tumor, and gold nanoparticles have been shown to work well for combination treatment using photothermal therapy and gene silencing. While a great deal of inorganic systems are still at the preclinical development stage, progress in nanomedicine is continually broadening the scope of possible applications. Artificial Intelligence (AI) is one of the key aspects of modern gene delivery research, with its role in engineering and optimizing vectors. The traditional approach to vector development has been to generate millions of variants or formulations of nanoparticles and then screen them by means of experiments, which is a time-consuming and expensive process and makes use of a lot of lab space. Nowadays, machine learning and deep learning algorithms can predict vector tropism, capsid stability, packaging efficiency, receptor binding affinity, intracellular trafficking, biodistribution, manufacturability, and immunogenicity, based on a large experimental dataset. Moreover, the field of vector engineering has been completely transformed by protein language models and generative AI platforms, which can now be used to design new viral capsids and synthetic delivery systems with ideal biological properties before lab testing. The process of AI-guided directed evolution has significantly shrunk the search space of experiments and has shifted the pace of finding vectors with increased tissue-specificity, immune evasion capabilities and therapeutic efficacy. These computational methods signal a paradigm shift from empirical screening of vectors to predictive, rational and more autonomous design of vectors. It should be acknowledged, however, that head-to-head, prospectively validated comparisons of AI-guided directed evolution against conventional directed evolution or rational design remain limited in the published literature; the speed and efficiency gains described above are therefore best regarded as promising but not yet definitively established, pending larger-scale benchmarking studies.

While general advances have been made, there are still major hurdles that prevent the use of current gene delivery systems for widespread applications. The limitations of the viral vectors are around the amount of cargo that can be carried, the immune response against them, the complexity of their manufacture and the cost of their production, while the non-viral vectors have lower transfection efficiency and are not very tissue-specific. Delivery to hard-to-reach organs like the brain, lungs, skeletal muscle or solid tumors remains a significant challenge. Moreover, these properties of long-term safety, repeated dosing ability, efficiency of intracellular trafficking and standardization of regulation are still far from being thoroughly investigated. These futuristic delivery systems are expected to combine synthetic biology, biomaterials engineering, nanotechnology, AI and precision medicine to create multifunctional vectors that will be able to deliver intelligent cargo, to sense the biological environment in real time, to adaptively target and to tailor therapeutic intervention to the individual. Therefore, improvements in gene delivery systems will continue to play a crucial role in the progress of gene therapy, not just in terms of the effectiveness of the therapy, but also in the broader clinical translation and global availability of next-generation genetic medicines.

### 2.4 Current clinical applications and limitations

The field of gene therapy has moved from an experimental idea to a clinically viable treatment option, radically changing the treatment paradigm for many inherited and acquired diseases. Significant progress in molecular genetics, vector engineering, genome editing, manufacturing technologies, and regulatory science has helped bring laboratory research into routine use in clinical practice. In the last 10 years, several gene therapy products have been approved in North America, Europe, Asia and elsewhere, highlighting the potential for single-dose, long-term therapeutic effects from genetic interventions (Martinez et al., 2022; He et al., 2024). Table 2 lists the clinical applications of gene therapy and associated limitations across major disease areas. Addiitonaly, Figure 4 demonstrate the clinical applications and therapeutic successes of gene therapy across major disease areas, highlighting approved treatments, emerging genome-editing strategies, and expanding precision medicine applications.

**TABLE 2:** Current clinical applications of gene therapy and associated limitations across major disease areas.

Disease category	Representative disease(s)	Gene therapy strategy	Representative approved/Clinical therapy	Major clinical outcomes	Current limitations
Inherited retinal disorders	RPE65-mediated retinal dystrophy, leber congenital amaurosis	AAV-mediated gene replacement	Luxturna (Voretigene neparvovec)	Restoration of retinal function, improved vision, long-term visual benefit	Limited to patients with specific mutations; retinal degeneration cannot be reversed at advanced stages; high treatment cost
Neuromuscular disorders	Spinal muscular atrophy (SMA)	AAV-mediated SMN1 gene replacement	Zolgensma (onasemnogene abeparvovec)	Improved motor function, prolonged survival, reduced ventilatory dependence	Age-dependent efficacy, immune responses to AAV vectors, extremely high manufacturing cost
Hemoglobinopathies	Sickle cell disease, β-thalassemia	CRISPR-based genome editing, lentiviral gene addition	Casgevy (exagamglogene autotemcel), lyfgenia (lovotibeglogene autotemcel), Zynteglo	Sustained fetal hemoglobin production, transfusion independence, reduced vaso-occlusive crises	Myeloablative conditioning required, long-term safety monitoring, limited accessibility
Primary immunodeficiency disorders	ADA-SCID, X-linked SCID	*Ex vivo* hematopoietic stem cell gene correction	Strimvelis and investigational lentiviral therapies	Restoration of immune function, reduced infection risk	Small patient populations, complex manufacturing, insertional mutagenesis concerns
Hemophilia	Hemophilia A and B	AAV-mediated gene replacement	Roctavian, hemgenix	Sustained coagulation factor expression, reduced bleeding episodes	Declining transgene expression, immune responses, inability to repeat AAV dosing
Hematological malignancies	B-cell acute lymphoblastic leukemia, diffuse large B-cell lymphoma, multiple myeloma	CAR-T cell therapy	Kymriah, Yescarta, Breyanzi, Carvykti, Abecma	High remission rates in refractory cancers	Cytokine release syndrome, neurotoxicity, individualized manufacturing, high treatment cost
Metabolic disorders	Aromatic L-amino acid decarboxylase (AADC) deficiency and other inherited metabolic diseases	AAV-mediated gene replacement	Upstaza and investigational therapies	Improved neurological and metabolic function	Limited long-term clinical data, disease-specific eligibility criteria
Emerging applications	Parkinson’s disease, Huntington’s disease, muscular dystrophy, cystic fibrosis, cardiovascular disorders	Gene replacement, genome editing, RNA-based therapeutics	Multiple ongoing phase I–III clinical trials	Promising preliminary efficacy and improved disease management	Delivery efficiency, off-target effects, long-term durability, regulatory approval pending
Sensorineural hearing loss	OTOF-related (otoferlin) genetic deafness	Dual AAV-mediated OTOF gene replacement	Otarmeni (lunsotogene parvec-cwha)	Restoration of auditory function in pediatric and adult patients with biallelic OTOF variants	Accelerated approval pending confirmatory trial data; candidacy limited to patients with preserved outer hair cell function and no prior cochlear implant

List current as of August 2026; representative rather than exhaustive.

**FIGURE 4:**
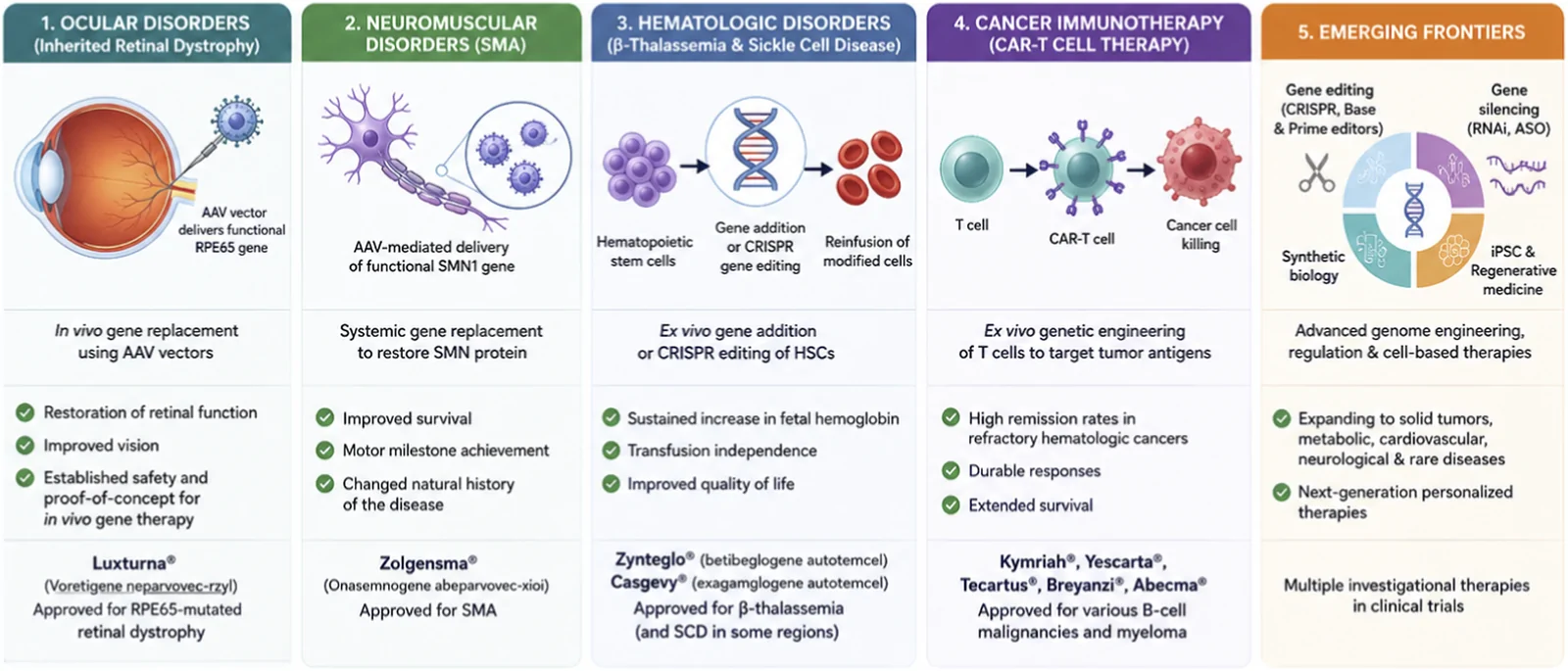
Clinical applications and therapeutic successes of gene therapy across major disease areas, highlighting approved treatments, emerging genome-editing strategies, and expanding precision medicine applications.

## 3 Artificial intelligence technologies for gene therapy

### 3.1 Machine learning, deep learning, natural language processing

AI has proved to be a game-changer in biomedical research, taking a new perspective on the approach of gene therapy discovery, development and clinical translation. Advances in high-throughput sequencing, multi-omics platforms, single-cell analysis, advanced imaging systems, electronic health records, and cloud-based computing power have led to the generation of unprecedented amounts of biological data, which traditional statistical methods are not sufficient to interpret (Sun et al., 2026). Chakrabarty and Mahajan (2024) comprehensively reviewed the expanding role of artificial intelligence (AI) in oncology imaging, emphasizing its transformative impact across the cancer care continuum through advanced deep learning and imaging analytics. The authors pointed out that convolutional neural networks (CNNs) were applied widely in cancer risk stratification, early detection, tumor diagnosis, genomic mutation prediction, response assessment to treatment, and survival outcome forecasting from multimodal imaging data (Figure 5). The review also highlighted the emerging role of AI in automated radiology reporting, which holds promise for faster reporting timelines and enhanced efficiency in complex clinical scenarios. Furthermore, the authors explored the increasing role of radiomics for the extraction and analysis of quantitative imaging features by machine learning algorithms to identify clinically relevant information not easily discernible by human observers. Biobanks were recognized as valuable resources for building strong AI models, as they offer high-quality, standardized datasets to train and validate algorithms. In addition to technological progress, the review focused on the key challenges related to clinical translation, such as model validation, generalizability, commercialization, ethical issues, and regulatory issues. The rise of generalist AI models was also noted as a potential area for creating more general clinical decision-support systems. Overall, the review illustrates the potential of AI-powered imaging analytics to improve precision oncology, including accurate diagnosis, tailored treatment planning, prognosis prediction, and evidence-based clinical action, offering valuable insights with applications that are highly relevant to the field of AI-driven precision medicine and future gene therapy innovations.

**FIGURE 5:**
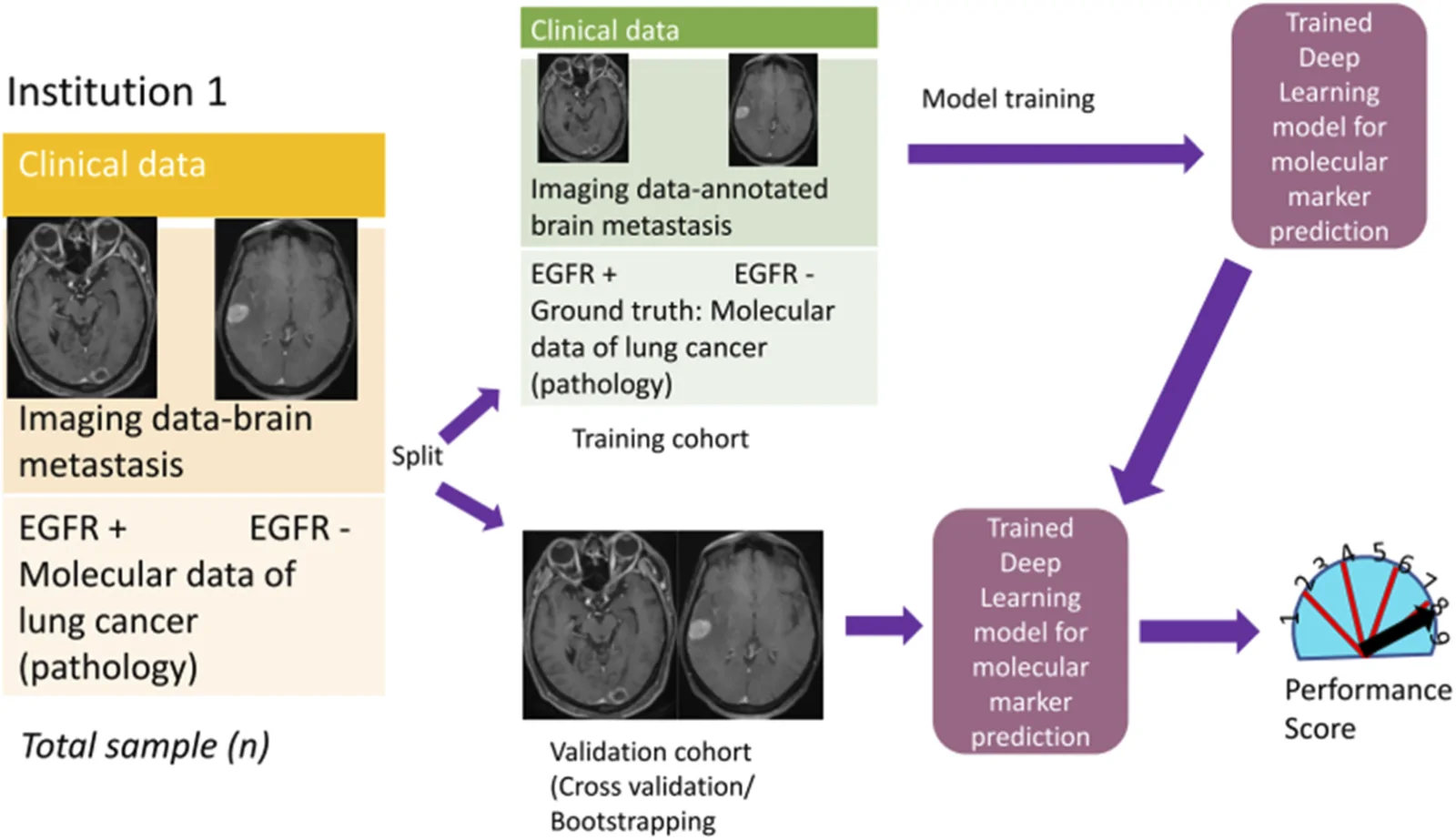
Training and validation of a deep-learning model (Chakrabarty and Mahajan, 2024).

Kausar (2025) designed a machine learning (ML) and Explainable Artificial Intelligence (XAI)-based model to forecast the prognosis (survival) of head and neck squamous cell carcinoma (HNSC) patients based on the expression profile of microRNA (miRNA) and clinical information. One of the biggest challenges of traditional AI applications in healthcare is a lack of interpretability, and the study addressed this by using XAI methods to detect the specific miRNAs that influence the prediction of patient survival. The proposed framework integrated miRNA expression data of 485 patients with HNSC and clinical information of 528 patients to build predictive survival models, which not only accurately classified patients into groups but also gave understandable explanations of their classification. The XAI model enabled healthcare providers to gain insights into the underlying biological mechanisms driving algorithmic decisions, enhancing confidence in the AI-aided diagnosis and treatment planning process. The XAI model helped healthcare providers identify disease-associated miRNA signatures, thereby gaining insights into the underlying biological mechanisms driving algorithmic decisions, which led to increased confidence in the AI-aided diagnosis and treatment planning process. The study illustrated how explainable ML can help to bring the predictive power of AI into the realm of clinical interpretation, paving the way for its wider use in oncology. In addition, the identified prognostic miRNAs could be used as biomarkers for patient stratification, prediction of patient survival, and precise therapeutic decision-making. In conclusion, this research highlights the significance of incorporating explainability in AI applications for biomedical fields, such as precision medicine and gene therapy, where clear and reliable identification of clinically relevant molecular biomarkers is crucial for the discovery of therapeutic targets, the selection of personalized treatments, and ensuring regulatory acceptance of AI-based clinical decision support systems.

### 3.2 Generative artificial intelligence, foundation models in biology, explainable artificial intelligence

Artificial Intelligence is undergoing a transformative era in recent times, marked by the rise of Generative Artificial Intelligence (Generative AI), Foundation Models, and Explainable Artificial Intelligence (XAI). But unlike traditional machine learning models, which mainly identify, predict and recognize patterns from known data sets, these cutting-edge AI paradigms have the power to generate new biological hypotheses, design therapeutic molecules, model complex biological systems and offer transparent decision-making frameworks (Albahri et al., 2023; Ye et al., 2026). Explainable Artificial Intelligence (XAI) is another transformative advancement that is assisting in making responsible utilization of AI in clinical settings (Xia et al., 2025). Bashir et al. (2026) introduced a complete system-level solution to build trustworthy digital healthcare integrating the Internet of Medical Things (IoMT), Large Language Models (LLMs), and Explainable Artificial Intelligence (XAI) in a single healthcare landscape. The authors viewed IoMT as the backbone of the continuous monitoring of patients and real-time collection of clinical data, and LLMs as the contextual reasoning engine for clinical documentation, patient communication, and intelligent decision-making support (Figure 6). Recognizing the need for more transparency, interpretability, and accountability in clinical decisions, the framework introduced XAI as a component designed to enhance the interpretability and transparency of black-box AI models. In addition, the research highlighted the importance of multimodal data fusion, which involves the fusion of physiological signals, clinical records, imaging data, and sensor data generated across the edge, cloud and distributed computing domains, aiding in the delivery of personalized healthcare. The authors showed how the intersection of IoMT, LLMs, and XAI can greatly improve healthcare interoperability, low-latency decision-making, and patient-centered care, while remaining robust and compliant with regulations. The proposed framework also emphasized the need for explainability and trustworthy AI to aid in the clinical uptake and the responsible use of intelligent healthcare systems.

**FIGURE 6:**
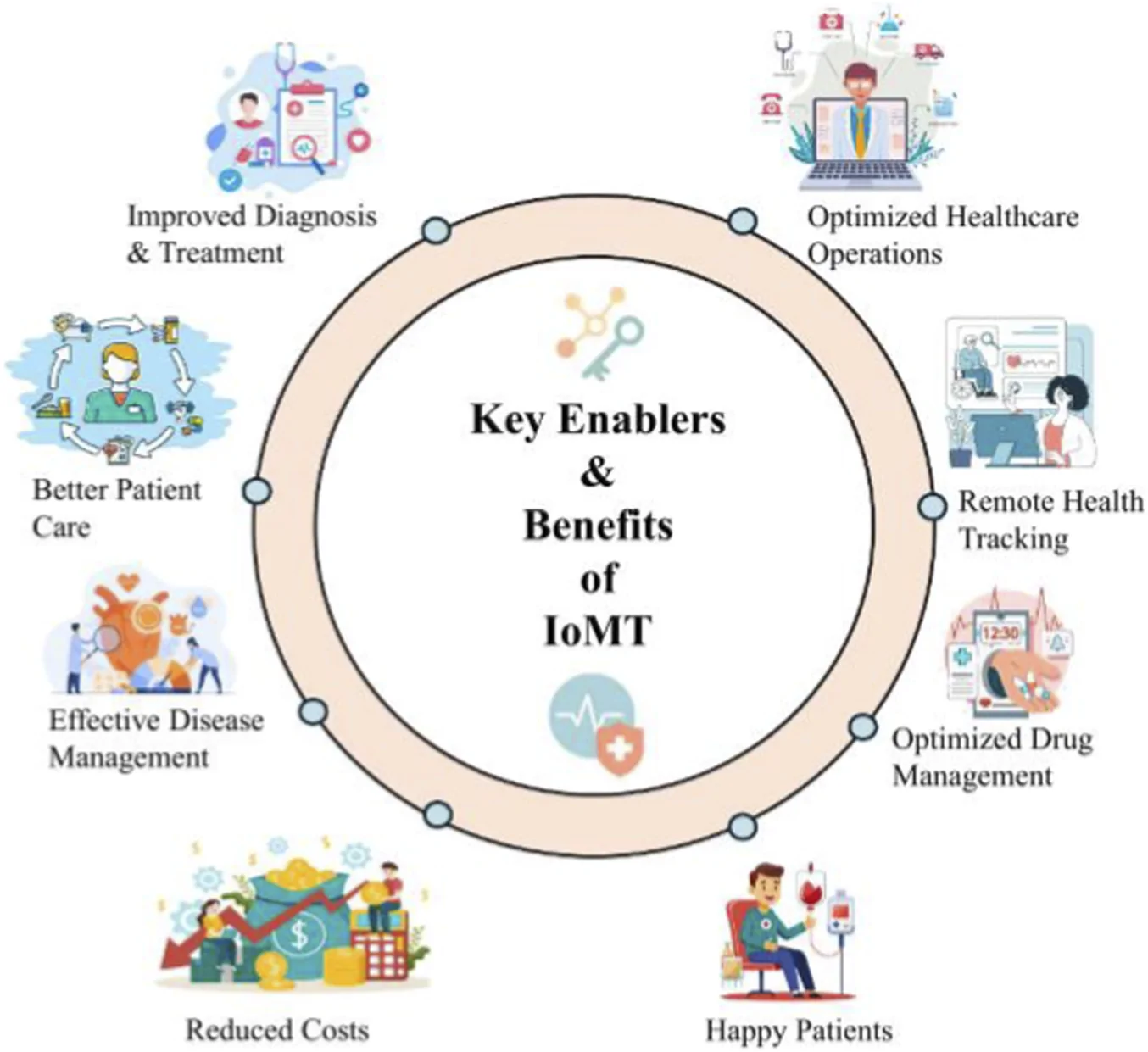
Key enablers and benefits of the IoMT in enhancing modern healthcare delivery (Bashir et al., 2026).

A systematic review was carried out by Joudar et al. (2022) that focused on the use of A.I. for the diagnosis, triage, and prioritized management for autism spectrum disorder (ASD) with special consideration of the role of genes. The authors systematically reviewed research published from 2017 to 2022 from the main scientific databases to assess the feasibility of using machine learning algorithms to combine clinical, genetic, and telemedicine data for diagnosis and prioritization of patients with ASD. The review included a breakdown of the existing literature, which could be divided into three groups–AI-assisted patient triage, prioritization of risk genes associated with ASD and telehealth-based electronic patient triage systems–and found that AI has shown significant promise in detecting genetic mutations, biomarker signatures, and toxic environmental factors linked to the progression of ASD. Moreover, machine learning methods were demonstrated to aid in early detection, disease severity, and patient stratification, enhancing the clinical pathway and effective utilization of resources. The authors also highlighted the need to combine genetic information with automated decision support systems for enhanced diagnostics and tailored treatment approaches. However, the review pointed to several challenges, such as a lack of readily available standardized datasets, variable methods for diagnostics, a lack of adequate multimodal biological data integration, and the importance of having strong EAI frameworks for enhancing clinical trust and adoption. The research identified an upcoming AI-powered approach for severity-driven triage and patient prioritization, leveraging intelligent decision-making methods, emphasizing the increasing significance of AI-aided genomic analysis and personalized medicine. These findings provide valuable insights for the development of AI-enabled gene therapy by supporting biomarker discovery, patient stratification, therapeutic prioritization, and precision clinical decision-making.

## 4 Artificial intelligence in therapeutic target discovery and genome engineering

One of the most important factors for successful development of gene therapy is the identification of the target(s) for treatment. The selection of a disease-associated gene is a huge challenge for scientists due to the huge genomic variation, gene regulation, epigenetic changes, and the interaction of environmental and cellular signaling pathways involved in genetic diseases (Khater et al., 2024; Suresh Kumar et al., 2026). Traditionally, therapeutic target discovery has been driven mainly by molecular biology experiments, candidate gene assays, genome-wide association studies (GWAS), functional genomics, and laboratory testing to validate, which are very costly, time-consuming, and labour-intensive. While they have made tremendous progress in understanding disease mechanisms, they often fall short of being able to incorporate the vast amount of multidimensional biological information produced by next-generation sequencing, transcriptomics, proteomics, metabolomics, epigenomics and single-cell technologies. Table 3 list artificial intelligence in therapeutic target discovery and genome engineering.

**TABLE 3:** Applications of artificial intelligence in therapeutic target discovery and genome engineering.

Application area	AI technologies	Representative models/Tools	Major contributions	Current challenges
Disease gene identification	Machine learning (ML), deep learning (DL), gradient boosting, random forest	DeepSEA, ExPecto, CADD	Identification of disease-causing genes and pathogenic variants from large-scale genomic datasets	Limited training data for rare diseases; population bias
GWAS interpretation	Deep neural networks, graph neural networks (GNNs), Bayesian learning	SNP prioritization frameworks, graph-based GWAS analysis	Prioritization of causal variants and disease-associated loci by integrating genomic and epigenomic information	Functional validation of predicted targets remains necessary
Multi-omics data integration	Multimodal AI, graph learning, deep neural networks	MOFA+, MultiVI, scAI	Integration of genomics, transcriptomics, proteomics, metabolomics, and epigenomics to identify therapeutic targets	Data heterogeneity and computational complexity
Single-cell data analysis	Variational Autoencoders, transformers, graph neural networks	scGPT, CellBERT, scVI	Identification of disease-associated cell populations, immune heterogeneity, and therapeutic targets	High computational cost and batch-effect correction
Functional genomics	Deep learning, transformer models	DeepSEA, Basenji, enformer, SpliceAI	Prediction of gene expression, chromatin accessibility, enhancer activity, and regulatory variants	Limited experimental validation of predictions
Biomarker discovery	Supervised ML, feature selection, explainable AI	Random forest, XGBoost, SHAP	Identification of diagnostic, prognostic, and predictive biomarkers for precision medicine	Clinical reproducibility and multicenter validation
Knowledge graph-based target discovery	Graph neural networks, knowledge graph AI	BioKG, hetionet, open targets	Discovery of novel relationships among genes, proteins, diseases, drugs, and pathways	Dependence on database completeness and quality
CRISPR guide RNA design	CNNs, RNNs, transformers	DeepCRISPR, DeepHF, DeepSpCas9, Azimuth	Prediction of guide RNA efficiency and optimization of genome-editing performance	Cell-type-specific variability and limited experimental datasets
Off-target prediction	Deep learning, ensemble learning	CRISPR-net, elevation, DeepCRISPR	Identification of potential off-target editing sites to improve genome-editing safety	Difficult prediction of rare off-target events *in vivo*
Base editing optimization	Machine learning, deep neural networks	BE-hive, DeepBaseEditor	Prediction of editing efficiency, bystander mutations, and editing windows	Diverse editor architectures require continuous model updates
Prime editing optimization	Deep learning, predictive ML	DeepPrime, PrimeDesign	Optimization of pegRNA design and prediction of prime-editing efficiency	Limited availability of large experimental training datasets
Protein engineering of CRISPR systems	Generative AI, protein language models	ESM, ProGen, ProteinMPNN	Design of novel nucleases with improved specificity, reduced immunogenicity, and expanded PAM compatibility	Experimental validation remains essential
Network-based therapeutic target discovery	Graph neural networks, systems biology	Protein–Protein interaction networks, STRING-based AI models	Identification of regulatory hubs, signaling pathways, and disease modules for therapeutic intervention	Biological network incompleteness
Experimental design Automation	Reinforcement learning, active learning, robotics	Autonomous laboratory platforms	Optimization of genome-editing experiments with reduced experimental iterations	Integration with laboratory infrastructure and regulatory validation

The CRISPR-Cas system is currently the most popular genome editing system due to its simplicity, programmability and versatility. With a short RNA molecule, the Cas nuclease identifies complementary sequences in the DNA and makes site-specific double-stranded breaks in the DNA, which are then repaired by endogenous cellular repair pathways, such as non-homologous end joining (NHEJ) or homology-directed repair (HDR). The key to the success of CRISPR-based therapy will be the ability to identify guide RNAs that are most effective at editing the target sequence, but will cause the least editing of the rest of the genome. The design of guide RNA was traditionally based on sequence heuristics and labor-intensive *in vivo* screening, consuming significant experimental resources and sometimes yielding low editing efficiency. The advent of AI has dramatically changed this process using machine learning and deep-learning algorithms that can learn the sequence determinants that dictate CRISPR activity directly from thousands of experimentally verified editing sequences.

Across the whole pharmaceutical pipeline, Qiao et al. (2025) thoroughly reviewed how the convergence of artificial intelligence (AI) and microfluidics can drive the entire drug research and development (R&D) process. The authors emphasized that microfluidic technologies can offer miniaturized, multiplexed, and high-throughput experimental platforms that produce high dimensional biological data, while artificial intelligence, such as machine learning (ML) and deep learning (DL), can help with speedy analysis and interpretation of this complex data and optimize it (Figure 7). The review found that the use of AI in microfluidic systems improves the various aspects of drug development, such as therapeutic target identification, compound screening, drug efficacy evaluation, drug toxicity assessment, drug manufacturing, and intelligent drug delivery. The use of AI-driven predictive models was demonstrated to enhance experimental design, boost screening efficiency, and minimize the time and expense of traditional drug discovery processes. In addition, the authors highlighted the promise of integrating microfluidics with AI to create automated, data-driven platforms that can aid precision medicine by providing personalized therapeutic assessment and real-time optimization of treatment strategies.

**FIGURE 7:**
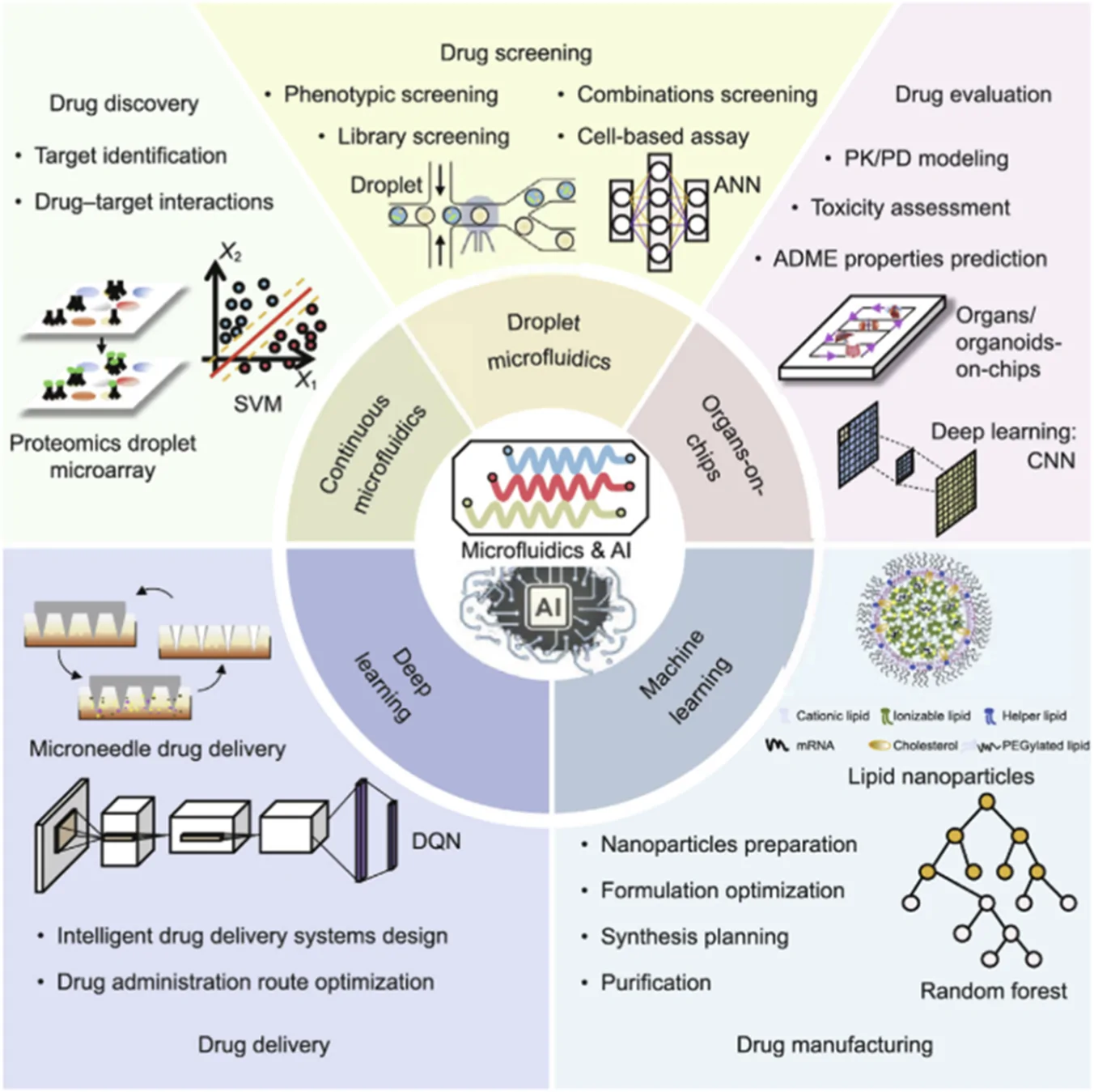
A summary of the synergy between AI and microfluidics throughout drug R&D. SVM: support vector machine; ANN: artificial neural network; CNN: convolutional neural network; DQN: deep Q-networks; ADME: absorption, distribution, metabolism, and excretion; mRNA: messenger RNA (Qiao et al., 2025).

Wang et al. (2024) suggested an intelligent treatment framework based on the combination of artificial intelligence (AI) and bioinformatics that would help to make diagnosis, prediction, and therapeutic management more effective for neuro-immune-digestive tract diseases, with special reference to gastrointestinal cancers. The study used several AI algorithms to analyse dietary patterns, isolate risk factors associated with diseases and forecast the likelihood of occurrence of gastrointestinal cancers, such as Self-Organizing Map (SOM) clustering, Isolation Forest, predictive machine learning models, and computer vision approaches. Bioinformatics and network pharmacology methods were also used to explore the molecular mechanisms of susceptibility gene mutations, immune signaling pathways, and the development of digestive tract malignancies, and molecular docking was used to identify therapeutic targets and candidate drugs. Additionally, computer vision technologies were integrated to improve the accuracy and efficiency of early cancer diagnosis, while addressing the shortcomings of conventional clinical reporting. The integrated AI framework also investigated the toxicological effects of betel nut consumption and its linkage with gastrointestinal carcinogenesis and neuropsychological disorders, allowing for a thorough understanding of the pathogenesis of these diseases. Lin et al. (2025) have created an Artificial Intelligence Raman Detection and Identification System (AIRDIS), a diagnostic platform based on AI and machine learning that could facilitate the rapid identification of antibiotic-resistant bacterial pathogens. This study was designed to develop a prediction algorithm for the presence of two key contributors to multidrug-resistant infection, methicillin-resistant *Staphylococcus aureus* (MRSA) and carbapenem-resistant *Klebsiella pneumoniae* (CRKP), based on label-free Raman spectral fingerprint data collected from clinical isolates. AIRDIS framework was trained and validated with a large dataset consisting of 988 *S. aureus* and 1053 *K. pneumoniae* isolates, which resulted in high prediction accuracy for the antimicrobial resistance profile. The machine learning model had high diagnostic performance with an accuracy of 88% (AUROC = 0.92) for MRSA and 87% (AUROC = 0.96) for CRKP, allowing the distinction between resistant strains and susceptible strains with a high degree of accuracy. The goal is to replace the time-consuming and tedious process of conventional microbiological testing, which can take multiple days to identify bacteria and their resistance profiles, with an AI-driven solution that can provide fast and accurate identification in a way that helps patients receive timely clinical care and targeted antibiotic treatment.

## 5 Artificial intelligence in gene therapy manufacturing

Along with therapeutic discovery and genome engineering, manufacturing processes that are robust, scalable, and economically sustainable are crucial for successful clinical translation of gene therapy (Wang et al., 2024). The manufacturing process is one of the most demanding steps in the gene therapy pathway, as it requires the production of very complex biological products such as viral vectors, nanoparticles that are not of viral origin, components for genome editing, messenger RNA, plasmid DNA and genetically engineered cells with strict regulatory and quality controls. Compared to traditional drug production, gene therapy production is highly biological in nature, and process reproducibility is very low, with high production cost and labor-intensive operations. Further, the scale-up of clinical trials and commercialisation of gene therapies has highlighted clinical manufacturing capacity and quality assurance, supply chain management and regulatory issues. Wani et al. (2024) introduced an interpretable AI model called BC^2^ (Breast Cancer Classification), which combines deep learning with ensemble machine learning for the purpose of breast cancer diagnosis with accuracy and transparency. The proposed hybrid approach exploits deep learning and ensemble learning techniques by using Convolutional Neural Networks (CNNs) for automatic feature extraction and the Light Gradient Boosting Machine (LGBM) for the classification task. The framework was evaluated on real-world breast cancer datasets, which showed an outstanding diagnostic performance with a 98.29% accuracy rate and high precision and recall, in comparison with several conventional machine learning algorithms (Figure 8). To overcome the “black-box” problem of AI in healthcare, the authors applied SHapley Additive exPlanations (SHAP) both globally and locally, generating interpretable explanations of the role of individual features in the prediction of models. The explainability module deepened the understanding and awareness of the clinical and biological logic behind each diagnosis, leading to a higher level of confidence in the use of AI in healthcare applications. The BC^2^ framework consisted of three integrated components: feature learning, diagnostic classification, and explanation generation, which formed a framework for making decisions in breast cancer management. The findings underscore the need for both predictive power and explainability when developing AI models that can be adopted in the clinic. The proposed framework offers valuable insights for precision medicine and AI-driven gene therapy, where interpretable machine learning could be used to aid in biomarker identification, patient stratification, therapeutic target prioritisation, and personalised treatment planning, along with transparency, regulatory compliance, and clinician confidence. We emphasize that this example concerns diagnostic classification rather than manufacturing itself; it is included here solely to illustrate the interpretable-AI architecture (feature learning, classification, SHAP-based explanation) that is directly transferable to manufacturing quality-control tasks such as in-process potency and purity assessment, where analogous explainability requirements apply.

**FIGURE 8:**
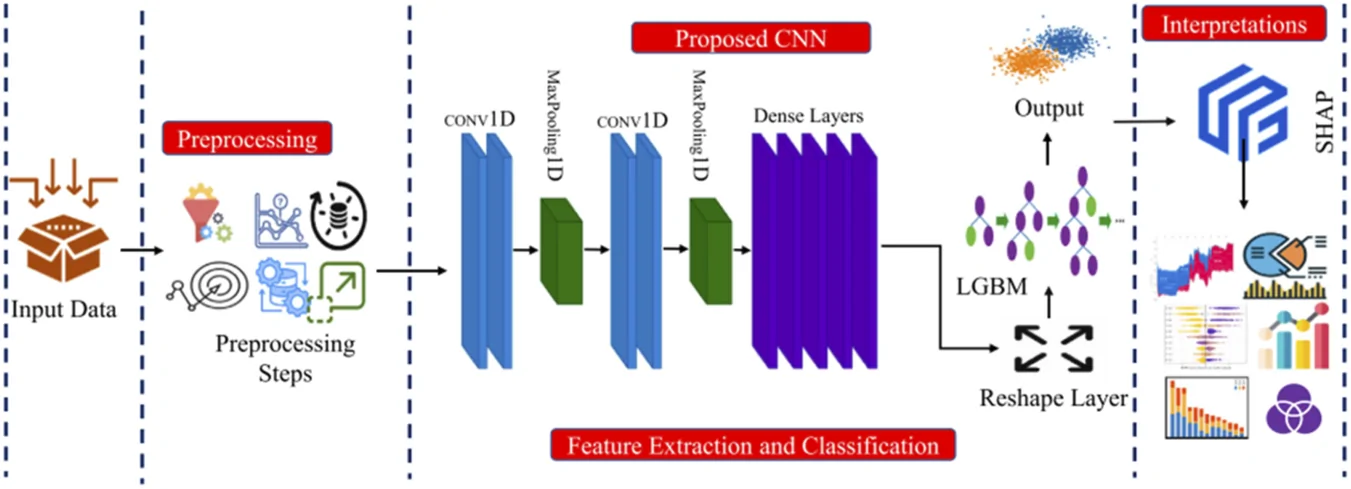
BC^2^ architecture (Wani et al., 2024).

By analyzing policy documents from the Overton database using the co-word network analysis technique, He et al. (2026) looked into the changing priorities in policy regarding Generative Artificial Intelligence (GenAI) and gene editing. The authors created co-word networks, in which key concepts were represented as nodes, using a dataset of 573 GenAI policies and 363 gene-editing policies to reveal thematic relationships, policy hotspots and long-term policy foci. The study proposed two new metrics: one-order and two-order h-cutoff metrics, to differentiate policy hotspots from strategic priorities and to compare the governance trends systematically between these two fast-growing technological fields. The analysis revealed 23 policy hotspots and four key policy foci for GenAI, and 14 policy hotspots and seven core policy foci for genome engineering, highlighting different priorities for regulatory and governance aspects between computational intelligence and genome engineering (Figure 9). The proposed methodology was found to be very repeatable, efficient and scalable for the analysis of large policy corpora, offering policymakers a non-subjective tool for tracking the technological trends and offering evidence to make informed decisions. As with the previous example, this study is not itself a manufacturing application; we cite it here as a methodological precedent for using AI-based text-mining to track regulatory and policy trends relevant to gene therapy manufacturing governance, rather than as evidence of AI applied to bioprocessing itself. A more directly relevant example is discussed below.

**FIGURE 9:**
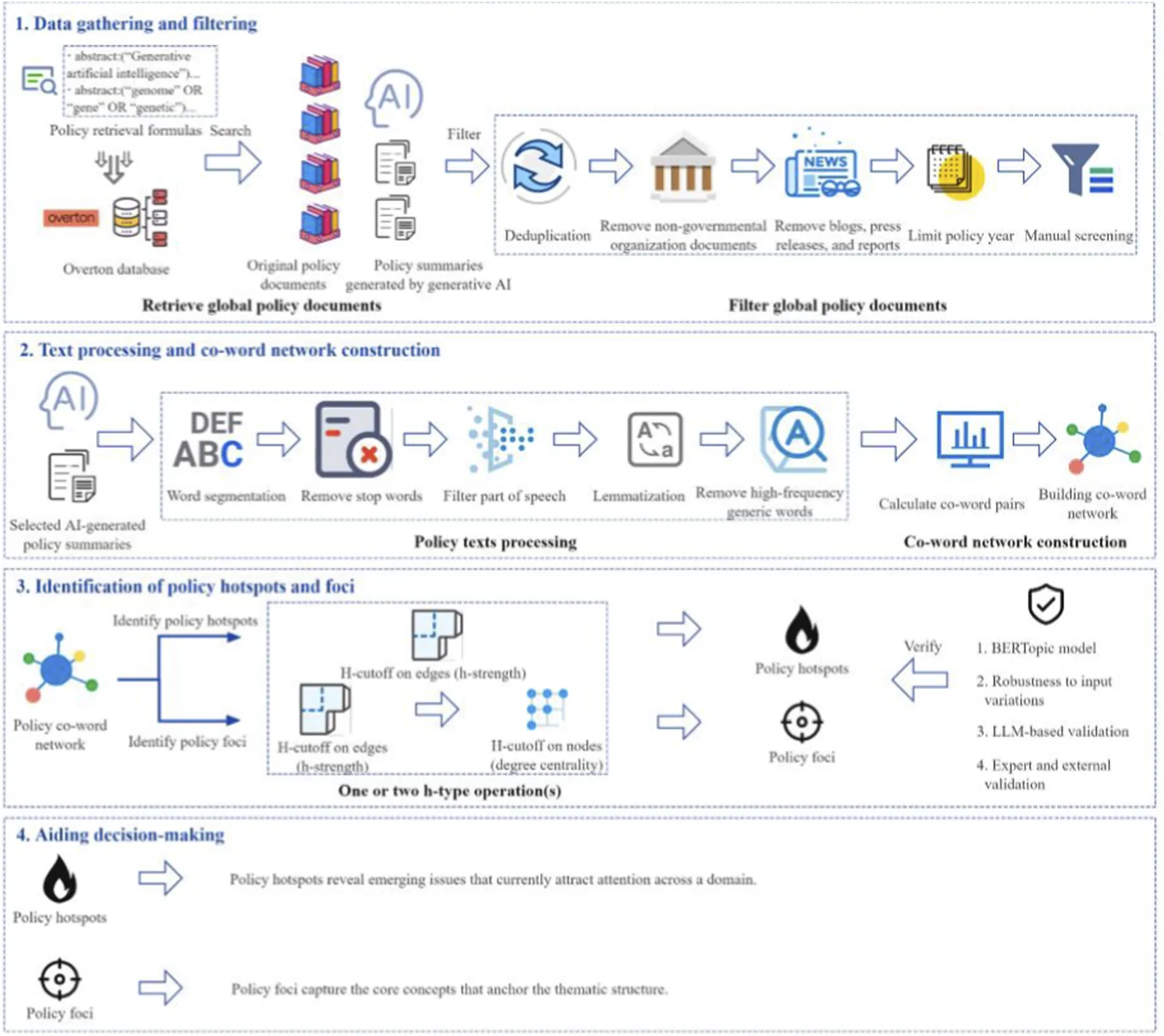
The methodological flowchart of the analysis (He et al., 2026).

Cortes-Briones et al. (2022) extensively covered the use of artificial intelligence (AI) and, in particular, deep learning (DL) in schizophrenia research, outlining the opportunities and the potential of using AI to tackle the complexity and diversity of schizophrenia. The authors pointed out that traditional statistical methods have proven to be too simplistic for modeling the high-dimensional, non-linear interactions between genetic, neurobiological, clinical, and environmental factors that play a role in schizophrenia. The use of deep learning algorithms, which incorporated multimodal datasets, was shown to achieve excellent results in various tasks such as disease classification, diagnosis, prognosis, prediction of disease relapse, characterization of symptoms, and assessment of treatment response. This example, too, is a diagnostic rather than a manufacturing application and is included only for its multimodal deep-learning methodology. A more directly relevant precedent is the AI-guided fermentation process regulation reported by Xu et al. (2025), in which machine learning models were applied to bioprocess data to reveal metabolic rewiring during gentamicin C1a overproduction and to guide real-time regulation of the fermentation process. Although developed for antibiotic bioproduction rather than gene therapy *per se*, this study demonstrates a manufacturing-relevant use case that is directly analogous to viral vector and plasmid DNA bioprocessing: AI-driven monitoring and control of upstream fermentation or cell-culture parameters to improve yield, consistency, and process robustness. Direct applications of AI to gene therapy-specific manufacturing steps, such as AAV capsid assembly efficiency, empty/full particle ratio control, and downstream purification chromatography, remain comparatively underrepresented in the published literature relative to diagnostic and policy-analytic applications of AI, and we identify this as a specific gap that future gene therapy manufacturing research should address.

## 6 Artificial intelligence in clinical translation

### 6.1 Patient selection and stratification

Successful gene therapy will require the creation of an effective therapeutic platform and the correct identification of patients who will benefit from such gene therapy interventions. Gene therapies are highly personalized and often target specific gene mutations, molecular pathways, or disease subtypes, whereas traditional pharmaceutical therapies are usually given to a large group of patients. Therefore, patient selection and patient stratification have emerged as key factors to success in therapy, long-term safety, clinical trials and utilization of healthcare resources (Sankaradass and Manish, 2026; Yu et al., 2026). Current methods of patient selection are largely dependent on genetic testing, clinical phenotyping, family history, level of disease severity and the experience of the clinician. These strategies have been helpful in the clinical application of several approved gene therapies, but they often miss the intricate molecular complexity of the large number of inherited and acquired diseases. Choudhary et al. (2025) discussed the applications of Artificial Intelligence (AI) in veterinary anatomy, which are ever-expanding and are increasingly becoming a part of educational, diagnostic and clinical decision-making for veterinary anatomy. The authors examined the role of AI technologies such as 3D modeling, VR, AR and AI-assisted image analysis in revolutionising the conventional approach to anatomy teaching and learning, offering students interactive and immersive learning experiences for enhanced anatomical visualization and understanding. In addition to education, AI-driven diagnostic systems were demonstrated to aid clinical examination and disease diagnosis by leveraging massive anatomical and imaging data, finding subtle structural abnormalities with greater precision and efficiency. The review also highlighted the potential of AI to assist with personalized veterinary care, based on data-driven diagnosis and treatment planning (Figure 10). The authors did note, however, that a number of challenges exist that could prevent broad adoption: reliance on large, high-quality annotated datasets, algorithmic bias, lack of model transparency, and data privacy and ethical governance concerns. In addition, too much use of virtual technologies could limit real anatomical studies and clinical experience. The study found that a balanced approach to the use of AI-assisted technologies and traditional teaching and clinical methods is key to maximizing their benefits. While primarily focused on veterinary medicine, the review highlights the potential of these cutting-edge developments in medical imaging analytics, explainable AI, intelligent decision support systems, and their impact on disease characterization, patient stratification, precision diagnostics, and the development of AI-driven precision medicine and gene therapy in the future. We note explicitly that this study does not evaluate gene therapy biomarkers, risk factors, or patient-selection criteria; it is included only for its treatment of AI-assisted imaging analytics and decision support, which are methodologically relevant but not a direct substitute for evidence on gene therapy patient stratification.

**FIGURE 10:**
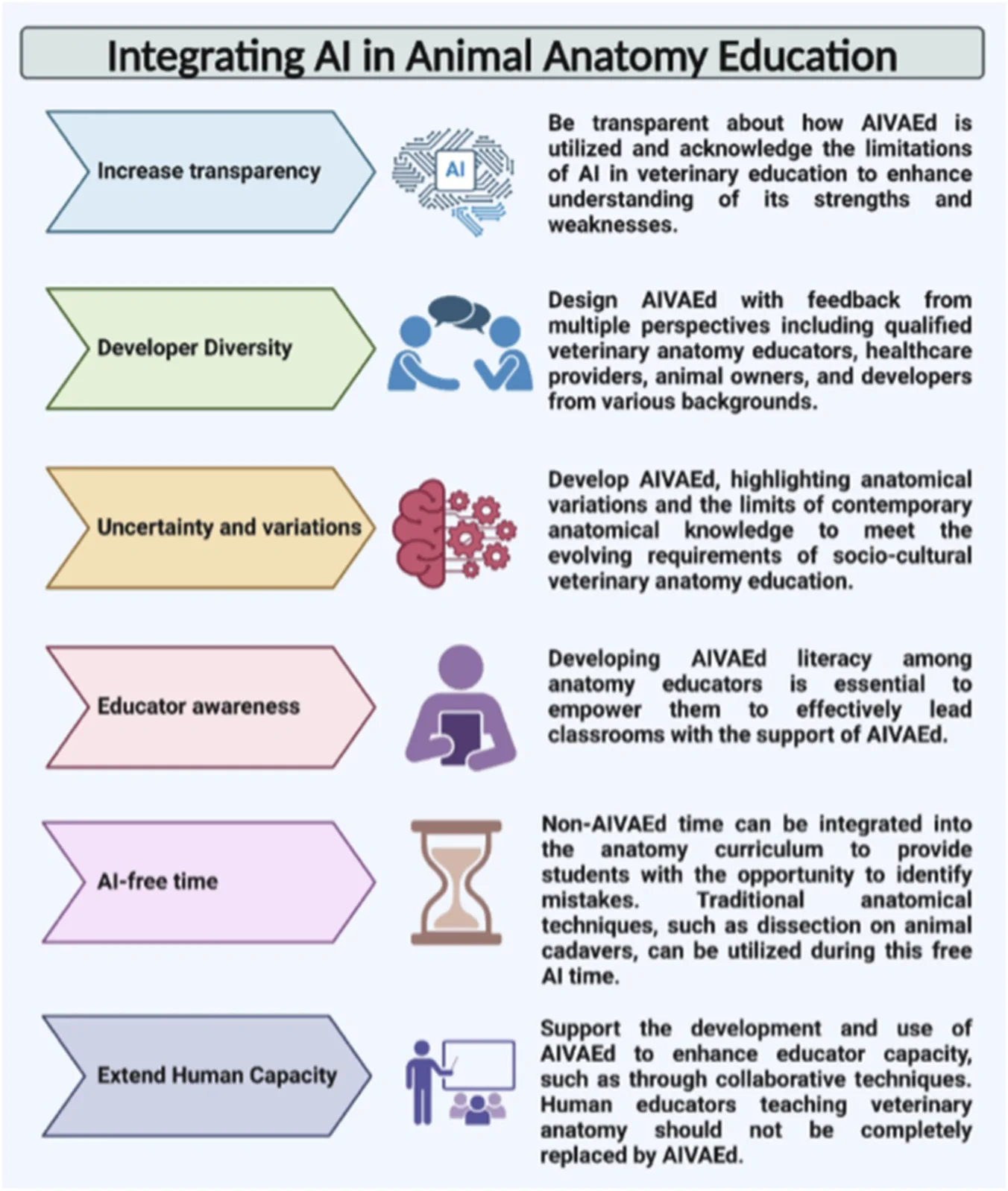
Usage of AI (Choudhary et al., 2025).

To enhance the diagnosis of liver disease, Suresh Kumar et al. (2026) designed an Intelligent Adaptive Neuro-Fuzzy Inference System (I-ANFIS) that combines artificial intelligence, adaptive learning, fuzzy logic and neural networks into a single system for liver disease diagnosis. The proposed model was built using a supervised machine learning approach that automatically optimizes fuzzy inference rules and membership functions for adaptive analysis of heterogeneous patient data, to deal with variations in liver disease phenotypes across different demographic groups. The I-ANFIS framework exhibited good modeling capabilities of complex nonlinear relationships within the clinical datasets, thanks to the learning ability of neural networks and the interpretability of fuzzy reasoning that led to improved diagnostic accuracy and reliability. Experimental results showed that it outperforms conventional deep learning methods, with an accuracy of 92%, precision of 93%, recall of 91%, F1 score of 92%, convergence speed, robustness, and computational efficiency. The authors also pointed to the potential framework for early disease detection, the development of context- and adaptive personalized treatment plans and the benefits for patient outcomes from using clinical decision support. The research demonstrates the increasing relevance of hybrid models, integrating predictive AI with explainable reasoning, for precision healthcare. While the I-ANFIS model was validated for liver disease diagnosis rather than gene therapy patient selection specifically, its architecture is directly relevant to the reviewer’s underlying question of whether such models evaluate biomarkers and risk factors: I-ANFIS explicitly incorporates heterogeneous clinical biomarkers and adapts its fuzzy membership functions to patient-level risk variation, a design principle that could analogously be applied to integrate genetic biomarkers, disease severity scores, and comorbidity risk factors into gene therapy candidacy models, though this application has not yet been demonstrated in the gene therapy literature.

### 6.2 AI-assisted clinical trial design

The clinical trial phase is one of the most challenging and expensive phases of gene therapy development, with significant financial investment, strict regulatory requirements, patient enrollment, and long-term safety monitoring. The clinical trials for gene therapy are even more complex than the traditional pharmaceutical trials due to the small patient population, the genetic diversity of the disease, the individual response to treatment, and the long-term follow-up, which is often needed lasting for many years. Many of the traditional approaches to clinical trial design, including fixed protocols, specific and strict eligibility criteria, and standard statistical methods, are unlikely to fully meet the needs of precision genetic medicine (Goertzel, 2007; Lindegger et al., 2022). As a result, many clinical trials fail to successfully translate into clinical practice due to delays in recruitment, insufficient statistical power, amendments to the protocol, high attrition rates, and rising development costs.

In a study, authors Khater et al. (2024) designed an explainable artificial intelligence (XAI)-based framework to examine how lifestyle factors affect obesity risk without relying on traditional body mass index (BMI) indicators, while employing machine learning models. The study used the Random Forest algorithm for obesity prediction with a prediction accuracy of 86.5% using behavioural and lifestyle characteristics. The authors implemented several model-agnostic explainability methods such as Shapley Additive exPlanations (SHAP), Partial Dependence Plots (PDP), and feature importance analysis to provide both global and individual-level explanations of model predictions, enhancing model transparency and making it easier to interpret in a clinical context. The explainable framework revealed that lifestyle factors most strongly correlated with obesity risk were meal frequency and technology use, highlighting the capability of XAI to reveal complex and nonlinear relationships, which are typically overlooked by traditional statistical analysis. The proposed model would offer clear insights into the contributions of these features, aiding in evidence-based clinical decision-making and personalized preventive healthcare strategies. The findings underscore the need for incorporating explainable AI with predictive machine learning to foster trust, explainability, and acceptance of AI systems within healthcare. The implications of these findings are particularly relevant to precision medicine and gene therapy, enabling better patient selection, understanding of the significance of biomarkers, prediction of therapeutic response, and personalization of treatments, and ensuring transparency, accountability, and regulatory trust in AI-driven clinical decision support systems. This example is drawn from obesity risk prediction rather than gene therapy trial design and is included solely to illustrate explainable-AI methodology (SHAP, PDP) that could, in principle, be repurposed to make gene therapy trial-eligibility models more interpretable to regulators and clinicians; it does not itself constitute evidence for AI-assisted gene therapy trial design. Xia et al. (2025) constructed an intelligent nanoplatform of H-MoS_2_/T-MoS_2_/Cu_3_Mo_2_O_9_ Z-scheme heterojunction for highly efficient and on-demand antibacterial therapy via synergistic nanotechnology and smart material engineering. The platform proposed was aimed at bringing together the phase-dependent properties of MoS_2_ with the loading of active components to facilitate charge separation and the production of reactive oxygen species (ROS) in an experiment verified with the help of density functional theory (DFT). Near-IR excitation of the nanoplatform could rapidly heat the microenvironment temperature up to 60 °C in just 6 min, and the generated ROS was significantly higher than that derived from conventional materials, and the bacteria *Escherichia coli* were nearly completely killed at low concentrations of the nanoplatform. One unique characteristic of the system was its ability to intelligently react to changes in the surrounding environment and autonomously switch between three antibacterial activities: Status protection, Real-time clearance, and dynamic attack, achieving precise, stimulus-responsive antibacterial activity. This adaptive behavior is evidence of the growing convergence of intelligent nanomaterials and intelligent design principles for next-generation therapeutic platforms inspired by artificial intelligence. While the study is about antibacterial applications, the intelligent decision-making capability, environmental responsiveness, and multifunctional therapeutic design offer valuable insights for precision medicine and gene therapy. The development of intelligent and adaptive biomedical therapies can be further benefited by the invention of such smart nanoplatforms, which could inspire future AI-assisted delivery systems that can enable controlled therapeutic activation, targeted biomolecule delivery, real-time monitoring of treatment progress and tailored intervention strategies. We acknowledge that this study centers on density-functional-theory-guided antibacterial nanomaterial design rather than artificial intelligence or clinical trial design, and its relevance to AI-assisted gene therapy trial design is indirect at best; it is retained only as an illustration of stimulus-responsive, autonomously adaptive therapeutic platforms, a design philosophy that parallels the adaptive clinical trial designs discussed below, and readers should not interpret it as AI-specific evidence.

Regulatory agencies, including the U.S. Food and Drug Administration (FDA) and the European Medicines Agency (EMA), have begun to formally adapt their frameworks to AI-assisted clinical development. The FDA’s Center for Biologics Evaluation and Research has issued draft guidance addressing the use of AI in the development of drugs and biological products, including gene and cell therapies, emphasizing a risk-based credibility assessment framework for AI models used in trial design, endpoint analysis, and manufacturing decisions. Adaptive and AI-informed trial designs, including Bayesian adaptive randomization and AI-assisted patient matching for rare disease cohorts, are increasingly discussed within expedited regulatory pathways such as accelerated approval and priority review vouchers, reflecting growing regulatory openness to computationally guided trial design for rare and ultra-rare genetic conditions where traditional randomized trial designs are often infeasible. Nonetheless, formal validation standards, transparency requirements, and post-approval monitoring expectations for AI models used in gene therapy trials remain under active development, and represent an important translational barrier addressed further in the following section.

### 6.3 Translational barriers from AI-Assisted discovery to clinical practice

Although the preceding sections illustrate substantial promise for AI across discovery, manufacturing, and clinical translation, the pathway from an AI-assisted finding to a clinically approved gene therapy remains comparatively underdeveloped and merits explicit discussion. First, regulatory validation of AI models used in gene therapy development is not yet standardized: unlike a fixed molecular entity, a machine learning model may be retrained as new data accrue, and regulators have not fully resolved how such “locked” versus “continuously learning” models should be validated, version-controlled, and monitored post-approval. Second, standardization across institutions remains limited, since AI models for vector design, patient stratification, or manufacturing quality control are frequently trained and validated on single-center or single-sponsor datasets, raising questions about generalizability before multicenter, prospective validation is undertaken. Third, manufacturing scalability is a persistent barrier: AI models that optimize small-scale bioprocess data, as in the fermentation example discussed in Section 5, must still be re-validated at commercial manufacturing scale, where batch-to-batch variability, raw-material sourcing, and facility-specific equipment can alter model performance. Fourth, economic considerations are substantial, as the infrastructure, computational resources, and specialized personnel required to build and maintain validated AI pipelines add to the already high cost of gene therapy development and may disadvantage smaller academic or biotechnology developers relative to large, well-resourced sponsors. Finally, long-term safety monitoring of AI-guided interventions, such as AI-designed vectors or AI-informed dosing, has not yet accumulated the multi-year follow-up data needed to confirm that computationally optimized products maintain the durability and safety profile of therapies developed through conventional approaches; the recently approved gene therapies listed in Table 2, most of which were developed without substantial AI input, currently provide the only long-term safety benchmarks against which future AI-assisted products will need to be compared.

## 7 Challenges and limitations

While AI has helped speed up therapeutic target discovery, genome engineering, vector optimization, manufacturing, the design of clinical trials, and post-marketing surveillance, there are still some challenges that prevent clinical reliability and widespread use of AI. These are due to the inherent complexity of biological systems, the apparent lack of computational power to simulate these systems, the lack of uniformity in healthcare data, regulatory uncertainty, and worries about transparency and safety of the algorithms. Unlike generic discussions of AI limitations, the challenges considered below are framed specifically around gene therapy: the rarity and heterogeneity of the patient populations that generate training data, the regulatory science that governs genetic medicines specifically, and the ethical stakes unique to heritable genome editing, rather than computing infrastructure or algorithmic performance in the abstract. Solving these challenges is crucial for bringing the full power of AI-driven gene therapy to fruition and for the sustainability of future gene therapy innovations that remain accurate, reproducible, equitable, and clinically trustworthy. The availability and quality of biomedical data are one of the most basic challenges that directly influence the performance and reliability of AI algorithms. High-throughput sequencing and sophisticated/platforms of analysis yield vast amounts of genomic, transcriptomic, proteomic, metabolomic, epigenomic, imaging and clinical data in the course of modern gene therapy research. But these datasets are often incomplete, lack annotations, have inconsistent formats, variability in measurements and are not biologically diverse. The vast majority of publicly available datasets have been generated under specific populations, experimental protocols and/or sequencing platforms, limiting their applicability to other ethnic groups, healthcare systems and disease conditions. Rare genetic disorders are even more challenging, as the populations of these disorders are small and large data sets necessary for developing robust machine learning models are not available. As a result, many AI models are developed based on base sets of data that are either limited or imbalanced, which makes them prone to overfitting and limits their accuracy when used on independent clinical samples.

Another constraint is that of regulatory ambiguities about AI in healthcare. Regulatory bodies around the globe are still grappling with regulations for AI-powered medical devices, genome editing, adaptive algorithms, and increasingly learnable computational systems. AI models can be updated continually as new data from clinical trials become available, a stark contrast to traditional medical products, which are relatively stable once approved. This dynamic behavior raises issues that are important for regulation, including: algorithm validation, version control, post-approval modification, algorithm transparency, and algorithm accountability. There is limited international harmonisation of AI regulation, which means uncertainty for developers aiming to commercialise an AI-powered gene therapy product internationally. The key challenge for future regulatory frameworks is to strike a balance between fostering innovation and comprehensive safety testing, while also adapting to the evolving landscape of AI technologies. Ethical issues are also significant challenges. The use of AI for gene therapy demands a vast amount of confidential genomic and clinical data, posing challenges in patient privacy, consent, data ownership, cybersecurity, and the misuse of genetic information. While international genomic databases are becoming more and more crucial for the construction of powerful AI models, it is important to ensure their secure sharing while respecting a variety of legal and ethical parameters that regulate patient confidentiality. The introduction of profound ethical questions regarding heritable genome editing, social justice, enhancement versus treatment, and responsible governance of technology are added by the application of germline genome editing. As the power of genome engineering grows, with the help of AI, it is vital to put in place comprehensive ethical control mechanisms to prevent misuse and safeguard trust in the technology.

## 8 Future opportunities

While existing AI applications have already achieved extraordinary results in the fields of drug discovery and clinical decision-making, the next decade will see an unprecedented combination of AI and synthetic biology, systems biology, robotics, nanotechnology, quantum computing, digital health and precision medicine. These interdisciplinary advances will take gene therapy from the realm of traditional genetic correction to a new level of intelligent, adaptive and personalized therapeutic ecosystems that continually learn from biological and clinical data. AI systems will no longer just serve as predictive analytical tools, but also as scientific partners that form their own hypotheses, design experiments, optimize therapeutic approaches, and participate in real-time clinical decisions. The potential for future advancements is significant in the ongoing development of biological FMs. As with natural language models, foundation models built from billions of sequences of genomes, transcriptomes, proteomes, cellular images, and biomedical publications are likely to yield common computational representations of biology. New models will incorporate DNA sequence, chromatin structure, RNA expression, protein interactions, epigenetic modifications, metabolomics and clinical phenotypes all into a comprehensive unified biological knowledge network. This comprehensive representation will allow us to accurately predict disease mechanisms, find new therapeutic targets, optimise genome editing strategies and design personalised therapies with few task-specific training. As these models grow in both size and biology, they can be the computational foundation for precision genomic medicine.

Precision genome editing is another field that could offer prospects, as AI will combine patient-specific genomic context, chromatin accessibility (Yu et al., 2026), DNA repair mechanisms, immune landscape, and disease stages into tailored editing approaches. Instead of generic CRISPR protocols, AI will suggest customised guide RNAs, programmable nucleases, delivery vectors and conditions for editing to suit each patient’s molecular make-up. New developments in base editing, prime editing, epigenome editing, RNA editing, and programmable gene regulation will enhance treatment options without disrupting the genome. AI-driven synthetic biology could also transform the creation of intelligent cellular therapies that would be able to detect disease-associated molecular signals and autonomously adjust therapeutic activity based on the biological conditions. Another promising prospect is the integration of quantum computing with AI. Still at a nascent stage, quantum computing has the promise to resolve complex optimization challenges such as problems with molecular interactions, protein folding, genomic network analysis and therapeutic design, which are too difficult to solve with classical computing architectures. When coupled with AI, quantum algorithms can potentially revolutionize fields of drug discovery, genome engineering, biomolecular simulation and the personalization of therapeutic optimization, offering entirely new possibilities in the realm of computational biology and precision medicine. It is important to note that quantum computing is fundamentally a hardware and computer-science discipline distinct from the machine learning and AI methods that form the core of this review, and its integration with gene therapy remains speculative: current quantum hardware has not yet demonstrated a validated advantage over classical high-performance computing for any gene therapy-relevant task. We include it briefly as a longer-term, exploratory direction rather than a near-term priority for AI-enabled gene therapy.

Realizing these opportunities depends on research priorities that are distinct from the present-day challenges outlined in Section 7: near-term priorities include prospective multicenter validation studies, standardized data-sharing frameworks, and regulatory science dedicated to adaptive AI models, whereas the longer-term opportunities described above (biological foundation models, AI-guided precision genome editing) represent directions for future investment once these foundations are in place. Achieving this will require sustained collaboration between computational scientists, clinicians, molecular biologists, ethicists, and regulatory agencies.

## 9 Conclusion

This review has traced how artificial intelligence is being applied across the gene therapy development continuum, from disease-gene and target identification through vector engineering, manufacturing, and clinical translation. Three principal messages emerge. First, the maturity of the evidence base is uneven: AI applications in diagnostics, imaging, and data analytics are comparatively well represented, whereas direct, validated applications to core gene therapy tasks, such as manufacturing process control and therapeutic target discovery, remain comparatively scarce, and this asymmetry should temper enthusiasm until it is addressed. Second, the principal barrier to clinical translation is not algorithmic performance but the translational infrastructure surrounding it: regulatory validation standards, multicenter data standardization, and long-term safety monitoring frameworks for AI-guided products are still being built, as discussed in Section 6.3. Third, realizing the field’s longer-term potential, including biological foundation models and AI-guided precision genome editing, will depend on sustained, multidisciplinary collaboration among computational scientists, biologists, clinicians, and regulators rather than on computational advances alone. AI is best understood, at this stage, as a powerful and rapidly maturing tool integrated throughout the gene therapy pipeline rather than as a substitute for the rigorous experimental and clinical validation that genetic medicines require.
